# The bidirectional relationship between emotional distress and subjective wellbeing among college students: a cross-lagged network analysis

**DOI:** 10.3389/fpsyg.2026.1863736

**Published:** 2026-07-06

**Authors:** Mengmeng Yuan, Wei Zhou, Bin Chen, Chang Hu, Joston Gary

**Affiliations:** 1 School of Marxism, Huainan Normal University, Huainan, Anhui, China; 2 Adamson University, Ermita, Manila, Philippines; 3 Physical Education of College, Jiangxi Normal University, Nanchang, Jiangxi, China; 4 Department of Management and Engineering, Linköping University, Linköping, Sweden

**Keywords:** college students, cross-lagged network model, emotional distress, mental health, subjective wellbeing

## Abstract

**Purpose:**

This study examined the bidirectional relationship between subjective wellbeing (SWB) and emotional distress (ED) among college students from a network perspective. It aimed to identify the cross-sectional network structure at two time points, identify the most central nodes, and explore bidirectional pathways between the two domains using cross-lagged network analysis.

**Methods:**

A two-wave longitudinal survey was conducted among college students from six universities in Jiangxi, Guizhou, and Jiangsu Provinces, China. Data were collected in December 2024 and December 2025 through the Wenjuanxing online platform. After screening and matching across waves, 1,354 students were retained for analysis. SWB was measured with the Index of Wellbeing Scale, and ED was measured with the Depression Anxiety Stress Scales-21. Cross-sectional networks were estimated at both time points, and a cross-lagged panel network model was used to examine cross-lagged relations over time.

**Results:**

The T1 and T2 networks had densities of 0.522 and 0.554, featuring predominantly positive within-domain and negative between-domain edges. At T1, “Life was meaningless” showed the highest strength, whereas at T2, “Hopeful” showed the highest strength. The cross-lagged network showed longitudinal predictive associations between SWB and ED from T1 to T2. Hopeful showed the strongest out-expected influence, 3.24, whereas Satisfied showed the strongest in-expected influence, 2.31. Network stability analysis supported the robustness of the centrality estimates.

**Conclusion:**

Network analysis revealed interconnected networks of SWB and ED among college students at both cross-sectional and longitudinal levels. The longitudinal findings indicate bidirectional predictive associations across a 1-year interval. These results highlight the value of a symptom-level approach and suggest that Hopeful and Satisfied may be informative nodes for future research on the temporal associations between positive and negative aspects of mental health.

## 1 Introduction

Mental health has become a serious concern among college students (Wang et al., 2020; Lipson et al., 2022; Chen et al., 2026). During college, students must adjust to academic demands, changing social relationships, greater independence, and uncertainty about the future (Zhao et al., 2023; Conley et al., 2020). These demands do not affect students only as broad states of distress or wellbeing. They may be expressed through specific cognitive, affective, and physiological responses, such as hopelessness, fear, agitation, tension, satisfaction, and perceived life meaning. A student may report anxiety-related symptoms while still retaining hope about the future, or may experience low life satisfaction without showing severe emotional symptoms. This pattern suggests that college student mental health should be examined not only through general levels of subjective wellbeing (SWB) and emotional distress (ED), but also through the specific components that connect positive and negative aspects of mental health (Nan et al., 2025). The systemic psychological toll of these demands is evident on a massive scale: across 62 meta-analyses encompassing over 8.7 million university students, the pooled prevalence reaches 35.41% for mild depression, 40.21% for mild anxiety, and 36.34% for stress (Paiva et al., 2025). These findings show that emotional distress affects a large proportion of college students and highlight the need to examine how specific elements of wellbeing and distress are organized in this population.

Within this interconnected web, subjective wellbeing (SWB) serves as a vital anchor for psychological adaptation (Galderisi et al., 2015; Martela and Ryan, 2023). As an individual’s cognitive and affective evaluation of life, SWB operates fundamentally as an active repertoire of specific positive components (Diener et al., 2003). The broaden-and-build theory explains the functional mechanism of these elements: experiencing positive states actively widens cognitive patterns, enabling individuals to construct lasting psychosocial resources (Fredrickson, 2004). For a college student navigating developmental challenges, this wellbeing materializes through granular, lived experiences—such as feeling hopeful about prospects, perceiving academic efforts as worthwhile, or remaining satisfied with current life circumstances (Liu et al., 2024). These distinct positive states drive academic functioning, foster daily adjustment, and actively buffer against subsequent depression risk. SWB is not only a general evaluation of life quality (Kelifa et al., 2021). It also reflects a set of specific positive components, such as hope, satisfaction, enjoyment, perceived reward, and perceived worth, that may shape students’ responses to academic and social demands (Zhou et al., 2025).

Coexisting within this same psychological ecosystem is emotional distress (ED), encompassing the subjective experience of aversive mental states (Xu and Huang, 2022). In the high-pressure academic context, this distress manifests directly as specific, disruptive symptoms—such as sudden panic, sustained agitation, or persistent fear during evaluative situations (Kashdan et al., 2015; Watson and Clark, 1984; Williams and Uliaszek, 2021). These concrete physiological arousals and affective reactions form the core architecture of depression, anxiety, and stress (Huang et al., 2025; Henry and Crawford, 2005; Coelho et al., 2026; Rezaei et al., 2025). Moving beyond mere internal discomfort, these specific distress components actively impair cognitive capacity and behavioral engagement (Awadalla et al., 2020). Their continuous accumulation depletes academic motivation, exacerbates concentration difficulties, and ultimately degrades grade point averages and overall satisfaction with university life (Pérez-Jorge et al., 2025; Rezaei et al., 2025; Liu and Wang, 2024). Consequently, ED operates as an interconnected cluster of aversive symptom-level experiences that systematically disrupt a student’s learning trajectory and broader adaptation.

The coexistence of these positive and aversive components is theoretically grounded in the dual-factor and complete state models of mental health. These frameworks conceptualize positive mental health and psychopathology as related but distinct dimensions of human functioning. For college students navigating complex academic environments, this dimensional approach captures a psychological reality: a student may experience acute emotional distress while simultaneously retaining specific positive psychological functions, or conversely, report minimal distress alongside an absence of wellbeing. Recent systematic evidence, particularly among Chinese college students, reinforces the need to assess these domains simultaneously (Hatun and Kurtça, 2026; Tang et al., 2024; Joshanloo, 2022). By framing SWB and ED as distinguishable domains, these models provide the essential conceptual foundation for mapping the intricate, cross-domain connections among specific symptoms and positive resources.

Building upon this dual-factor foundation, empirical research has established a robust bidirectional, longitudinal relationship between these two domains. Over extended developmental periods, accumulated emotional distress operates as a critical antecedent to diminished psychological flourishing; specifically, elevated depressive and stress symptoms consistently forecast subsequent declines in life satisfaction and overall wellbeing (Buecker et al., 2023). Conversely, robust positive psychological functioning serves an active preventative role (Margraf et al., 2020). Stabilizing and improving an individual’s subjective wellbeing systematically predicts lower future vulnerability to depression and negative affect (Zhao et al., 2021). These studies confirm that wellbeing and distress function as mutually interacting global constructs, demonstrating that positive adaptation actively mitigates future psychopathology, just as prolonged distress erodes future flourishing.

Positive mental health and psychological distress are related but distinct dimensions (Keyes, 2005; Greenspoon and Saklofske, 2001). This distinction is especially relevant for college populations. A student may report minimal emotional distress yet show low wellbeing, or may experience academic and emotional strain while maintaining positive psychological functions such as hope, satisfaction, or perceived meaning (Antaramian, 2015; Xiao et al., 2021). Evidence from Chinese college students and recent systematic work further supports the value of assessing both positive mental health and distress simultaneously (Magalhães, 2024; Eklund et al., 2010).

Although previous longitudinal studies robustly support reciprocal associations between SWB and ED, most evidence still relies on variable-level models. These traditional analytical approaches typically reduce each broad domain to a latent factor or a sum score, operating on the assumption that individual survey items function as interchangeable indicators of a unified common cause (Serafim et al., 2025; Rezaei et al., 2024). However, this assumption is overly restrictive for understanding college student mental health because SWB and ED are profoundly heterogeneous domains. Specific psychological elements—such as a cognitive sense of life meaninglessness, future-oriented hope, broad life satisfaction, or physiological agitation and fear—each occupy functionally distinct positions within the mental system. Consequently, while traditional sum-score models can effectively demonstrate that global wellbeing and distress are correlated, they cannot, in principle, reveal which specific item-level components link the two domains or drive their interactions (Santini et al., 2022; Kairys et al., 2025; Yang et al., 2026).

Network analysis offers a complementary approach to examining heterogeneity in SWB and ED by representing individual indicators as interconnected nodes. This framework estimates conditional relations among specific components and distinguishes within-domain connections from cross-domain links (Keyes et al., 2020; Cheng et al., 2025; Xiang et al., 2025). A growing body of research has already applied network methods to the study of wellbeing, positive psychological resources, depression, anxiety, and psychological distress (Su et al., 2023). Previous studies have mapped networks of psychopathology and wellbeing, examined item-level links among daily stressors, psychological capital, and distress, and evaluated network changes following positive psychology interventions (Campbell and Osborn, 2021; Pan et al., 2024; Tejada-Gallardo et al., 2022). These studies provide an important foundation for examining how specific components of positive psychological functioning and emotional distress are interconnected.

Despite these advances, much of the existing network evidence remains cross-sectional, whereas longitudinal studies have often relied on global construct scores (Lei et al., 2025; Marcos-Escobar et al., 2025; Hu et al., 2025; Błoch and Misiak, 2026; Carvalho et al., 2022; Hu et al., 2025). Consequently, it remains unclear whether specific components of SWB prospectively predict particular ED symptoms and whether specific ED symptoms predict later components of wellbeing. Longitudinal item-level evidence addressing these reciprocal associations among college students remains limited (Dai et al., 2026; Cao et al., 2025; Zhou et al., 2022). This gap highlights the need for an approach that can extend existing network research from contemporaneous associations to prospective component-level relationships.

Cross-lagged panel network (CLPN) analysis provides such an approach. By estimating component-to-component predictive pathways across waves, CLPN analysis can examine whether specific SWB components predict later ED indicators and whether specific ED components predict later wellbeing indicators, after accounting for the broader baseline network (Zhang et al., 2024). It also identifies nodes with relatively strong outgoing or incoming predictive connectivity, thereby indicating which components carry greater prospective information within the longitudinal network. Nevertheless, a two-wave design cannot establish causal feedback loops, rule out unmeasured third variables, or fully capture complex developmental dynamics. Accordingly, the present study extends the growing network-based literature by using cross-sectional and cross-lagged network analyses to examine SWB–ED associations among college students. Specifically, it aims to map the item-level organization of SWB and ED at two time points, estimate T1-to-T2 predictive pathways between specific wellbeing and distress components, and identify nodes with relatively strong outgoing or incoming predictive connectivity.

## 2 Materials and methods

### 2.1 Participants and procedure

This study used a regional convenience sampling design with voluntary participation. Participants were recruited from six universities in Jiangxi, Guizhou, and Jiangsu Provinces, China. Therefore, the sample should be understood as a regional sample of college students rather than a nationally representative sample of Chinese college students. At each university, teachers assisted with recruitment by informing students about the study’s purpose and importance, then sharing the survey link via class-based online channels. Students decided for themselves whether to participate. We collected data at two time points through the Wenjuanxing online survey platform: December 2024 (T1) and December 2025 (T2).

To enter the study, participants had to meet the following inclusion criteria. First, they had to be currently enrolled as students at one of the six participating universities. Second, they had to agree to the informed consent form before starting the survey. Third, they had to complete the online questionnaire independently. Fourth, each participant was assigned a randomly generated tracking code by the online survey system for cross-wave matching. For the final longitudinal analyses, participants also had to provide valid data at both time points and be successfully matched across the two waves. The online survey system randomly generated the tracking code and did not allow participants to generate their own. No names or full identification numbers were collected. The tracking information was stored in the password-protected survey backend, accessible only to authorized members of the research team. The code was used solely to match T1 and T2 responses.

At T1, 1,668 students completed the survey. We excluded questionnaires with missing values, completion times shorter than 60 s, invariant response patterns across scale items, duplicate responses, or invalid tracking information. After screening, 1,597 (95.7%) valid cases remained. At T2, 1,417 (85.0%) students completed the follow-up survey. We applied the same exclusion criteria and retained 1,354 (81.2%) valid cases. The final analytic sample, therefore, included 1,354 students who provided valid responses at both time points.

We stored all data in encrypted form throughout the study and destroyed the original linkage information immediately after the matching process. This procedure ensured that the research team could not trace any response back to a specific individual. All participants signed informed consent before completing the questionnaire. The study was conducted in full compliance with the Declaration of Helsinki and local legal requirements, and was approved by the Ethics Committee of Jiangxi Normal University (IRB-JXNU-PEC-20240402).

### 2.2 Measures

#### 2.2.1 Subjective wellbeing

This study measured SWB with the Index of Wellbeing Scale (IWB) developed by Campbell (1976). The IWB contains 9 items and covers two dimensions: the Overall Affect Index (8 items) and Life Satisfaction (1 item). A sample item is “Life has been so good to me.” Participants rated each item on a 7-point Likert scale from 1 (strongly disagree) to 7 (strongly agree). Following the original scoring procedure, this study calculated the WBIS score by adding the mean score of the Overall Affect Index (weight 1.0) to the Life Satisfaction score (weight 1.1). Total scores ranged from 2.1 to 14.7, and higher scores indicated greater SWB. In the present study, the Cronbach’s alpha coefficients for the WBIS were 0.926 at T1 and 0.918 at T2.

#### 2.2.2 Emotional distress

This study measured ED with the Depression Anxiety Stress Scales-21 (DASS-21) developed by Lovibond and Lovibond (1995). The DASS-21 contains 21 items and includes three dimensions, Depression, Anxiety, and Stress, with 7 items in each dimension. A sample item is, “I felt I was close to panic.” Participants rated each item on a 4-point Likert scale from 0 (did not apply to me at all) to 3 (applied to me very much or most of the time). For this study, the DASS-21 served as an indicator of overall ED. Higher scores indicated higher levels of emotional distress. In the present study, the Cronbach’s alpha coefficients for the DASS-21 were 0.927 at T1 and 0.933 at T2.

### 2.3 Data analysis

Data analysis proceeded in several steps. SPSS 26.0 was used to calculate descriptive statistics and Pearson correlations for the main study variables. Harman’s single-factor test was also conducted to examine common method bias (Baumgartner and Weijters, 2021). Longitudinal measurement invariance was tested in Mplus 8.3 separately for subjective wellbeing and emotional distress using the item-level indicators included in the subsequent network analyses. All nine IWB items and all 21 DASS-21 items were included at both T1 and T2. The same measurement structure was specified across the two waves for each measure. The invariance analyses followed a sequential procedure. Configural invariance, M1, was first examined without equality constraints across time. Metric invariance, M2, was then tested by constraining the corresponding item loadings to equality across T1 and T2. Scalar invariance, M3, was tested by additionally constraining the corresponding item intercepts to equality across time. Residual correlations were allowed only between corresponding items across T1 and T2 to account for item-specific temporal stability. No residual correlations between non-corresponding items were specified. Longitudinal invariance was supported when ΔCFI was ≤ 0.01 and ΔRMSEA was ≤ 0.015 (Chen, 2007).

The network analyses were conducted in R Studio. In the cross-sectional network analysis, the items of subjective wellbeing and emotional distress were treated as nodes. Separate networks were estimated for T1 and T2 with the qgraph package (Epskamp et al., 2012). The EBICglasso procedure was used to regularize the networks and remove weak edges (Johal and Rhemtulla, 2021). The correlation method was set to Cor, and pairwise missing values were excluded. The estimated networks were weighted and signed. The EBICglasso tuning parameter was set to gamma = 0.5. For centrality output, normalized centrality measures were reported. A Spring layout was adopted to maintain consistent node positions across the two time points. Strength was used as the primary centrality index for interpreting the cross-sectional networks. Closeness was also calculated but treated only as a descriptive, exploratory metric due to concerns about its stability and interpretability in psychological networks (Borsboom et al., 2021).

The cross-lagged panel network (CLPN) model was estimated using node-wise regularized linear regression with an LASSO penalty in the glmnet package. The LASSO model was specified by setting alpha = 1 (Rhemtulla et al., 2017). In this model, each T2 item was regressed on all 30 T1 items. The predictor matrix included all T1 SWB and ED items, and the outcome variables were the corresponding T2 items. Because each item’s own T1 score was included in the predictor matrix, autoregressive effects were estimated and controlled for in all node-wise regressions. Cross-lagged effects were estimated simultaneously by examining whether each T1 item predicted other T2 items. The model was estimated with standardized predictors (standardize = TRUE). To improve reproducibility, the random seed was set to 100 before each node-wise cross-validation. The optimal regularization penalty parameter, lambda, was selected using 10-fold cross-validation based on the deviance statistic, and the conservative one-standard-error rule, lambda. 1se, was used to obtain a more parsimonious network and reduce the risk of false-positive edges. Standardized regression coefficients from the node-wise LASSO models were used to construct the directed cross-lagged network. Node importance in the longitudinal network was evaluated using in-expected influence and out-expected influence. Out-expected influence reflects the extent to which a T1 node predicts other nodes at T2, whereas in-expected influence reflects the extent to which the T1 network predicts a T2 node. Network stability was examined with the bootnet package. A case-drop bootstrap procedure was used to assess the stability of the centrality estimates (Epskamp et al., 2016).

## 3 Results

### 3.1 Common method variance analysis

Harman’s single-factor test was conducted as a limited preliminary assessment of potential common method variance. All items from each wave were subjected to exploratory factor analysis, and the variance explained by the first unrotated factor was examined. At T1, the first factor accounted for 37.42% of the total variance, whereas at T2 it accounted for 39.27%. Although neither value exceeded the conventional 40% threshold, Harman’s single-factor test provides only a limited assessment and cannot rule out the presence of common method variance (Aguirre-Urreta and Hu, 2019). These findings should therefore be interpreted cautiously.

### 3.2 Demographic information

A total of 1,354 college students were included in the current study. The sample comprised 578 males (42.69%) and 776 females (57.31%). Regarding registration location, 632 participants (46.68%) were from rural areas, while 722 (53.32%) were from urban areas. In terms of educational background, the majority of the sample consisted of undergraduate students (*n* = 1,151, 85.01%), followed by master’s students (*n* = 163, 12.04%) and doctoral students (*n* = 40, 2.95%).

Preliminary analyses (e.g., independent samples *t*-tests and one-way ANOVAs) were conducted to examine potential demographic differences in the main study variables. The results indicated no significant differences in SWB and ED by gender, registration location, or educational level (all *p* > 0.05). Consequently, these demographic characteristics were not included as covariates in the subsequent cross-lagged panel network analyses (see Table 1).

**Table 1:** Demographic characteristics and group differences.

Demographic characteristic	*n*	Proportion	T1SWB	T2SWB	T1ED	T2ED
			*t*/*F*(*p*)	*t*/*F*(*p*)	*t*/*F*(*p*)	*t*/*F*(*p*)
Gender			0.968 (0.33)	0.630 (0.53)	−0.996 (0.32)	0.019 (0.98)
Male	578	42.69%				
Female	776	57.31%				
Registration location			1.241 (0.21)	1.087 (0.28)	−1.168 (0.24)	−0.591 (0.55)
Rural	632	46.68%				
City	722	53.32%				
Education			2.804 (0.06)	2.247 (0.11)	1.737 (0.18)	1.486 (0.23)
Bachelor	1,151	85.01%				
Master	163	12.04%				
Doctor	40	2.95%				

Unpaired *t*-test and ANOVA results (two-tailed test).

### 3.3 Measurement invariance analysis

Configural, metric, and scalar invariance were tested to examine the longitudinal comparability of SWB and ED across T1 and T2. The configural models showed acceptable fit for both constructs, indicating that the same factor structure was supported across T1 and T2. The subsequent metric and scalar models also met the recommended criteria for changes in fit indices. These findings indicate that the two measures remained sufficiently comparable across the two waves and therefore justified the subsequent longitudinal analyses (see Table 2).

**Table 2:** Measurement invariance of emotional distress and subjective wellbeing.

Variable	Model	*χ* ^2^	df	CFI	TLI	SRMR	RMSEA	Model comparison	ΔCFI	ΔRMSEA
SWB	M1	118.935	125	0.998	0.999	0.011	0.012			
	M2	122.884	133	0.998	0.998	0.014	0.010	M2-M1	0	−0.002
	M3	189.435	148	0.989	0.990	0.021	0.018	M3-M2	−0.009	0.008
ED	M1	803.608	783	0.999	0.999	0.018	0.004			
	M2	819.890	801	0.999	0.999	0.019	0.005	M2-M1	0	0.001
	M3	833.357	819	0.998	0.999	0.019	0.005	M3-M2	−0.001	0.000

M1 = configural invariance model. M2 = metric invariance model. M3 = scalar invariance model. Corresponding residuals were allowed to correlate across T1 and T2.

### 3.4 Correlation analysis

The skewness values ranged from −0.635 to 0.457, and the kurtosis values ranged from 0.228 to 0.999. These values were within acceptable ranges (absolute skewness < 2, absolute kurtosis < 7; Kline) (Lei and Lomax, 2005), indicating that the data approximated a normal distribution (see Table 3).

**Table 3:** Mean, standard deviation, and correlation matrix of subjective wellbeing and emotional distress.

Variable	M	SD	T1 SWB	T2 SWB	T1 ED	T2 ED	Skewness	Kurtosis
T1 SWB	9.68	1.86	1				−0.07	−0.11
T2 SWB	9.79	1.79	0.609^**^	1			0.01	−0.16
T1 ED	0.97	0.64	−0.502^**^	−0.487^**^	1		0.57	−0.32
T2 ED	1.01	0.66	−0.464^**^	−0.539^**^	0.721^**^	1	0.49	−0.54

**p < 0.01.

Pearson correlation analyses indicated that SWB at T1 was positively correlated with SWB at T2 (*r* = 0.609, *p* < 0.01), and ED at T1 was positively correlated with ED at T2 (*r* = 0.721, *p* < 0.01). At the same time points, SWB was negatively correlated with ED at both T1 (*r* = −0.502, *p* < 0.01) and T2 (*r* = −0.539, *p* < 0.01). Across different time points, T1 SWB was negatively correlated with T2 ED (*r* = −0.464, *p* < 0.01), and T1 ED was negatively correlated with T2 SWB (*r* = −0.487, *p* < 0.01).

### 3.5 Cross-sectional network analysis

**Figure 1:**
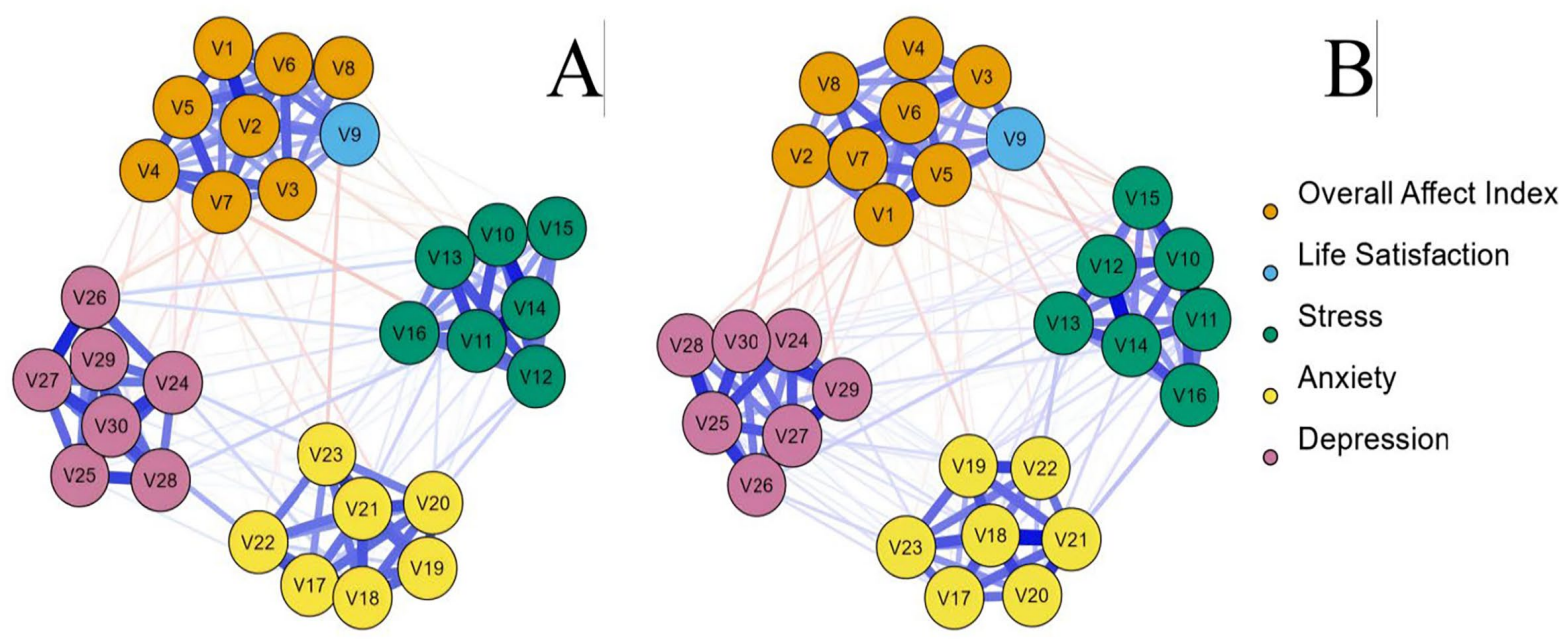
Cross-sectional network analysis. A = T1 network; B = T2 network. V1 = Hopeful, V2 = Enjoyable, V3 = Interesting, V4 = Friendly, V5 = Rewarding, V6 = Worthwhile, V7 = Full, V8 = Brings out the best in me, V9 = Satisfied, V10 = Hard to wind down, V11 = Over-react, V12 = Drain nervous energy, V13 = Agitated, V14 = Difficult to relax, V15 = Intolerant, V16 = Touchy, V17 = Dryness of my mouth, V18 = Breathing difficulty, V19 = Trembling, V20 = Worried fool, V21 = Panic, V22 = Heart rate increase or missing a beat, V23 = Scared, V24 = Lack of positive felling, V25 = Absence of initiative, V26 = Nothing to look forward, V27 = Down hearted and blue, V28 = Unable to be enthusiastic, V29 = Useless, V30 = Life was meaningless.

The cross-sectional network structures of SWB and ED at T1 and T2 are illustrated in Figure 1 (Panel A for T1 and Panel B for T2). Both networks comprised 30 nodes, including 9 SWB items (labeled V1-V9) and 21 ED items (labeled V10-30).

The T1 network had 227 non-zero edges out of 435 possible edges, yielding a network density of 0.522. The T2 network had 241 non-zero edges out of 435 possible edges, yielding a network density of 0.554. To examine whether the two networks differed significantly, we conducted a Network Comparison Test, NCT. The results showed no significant difference in edge weights between the T1 and T2 networks, *p* = 0.19. The difference in global strength was also not significant, *p* = 0.85.

**Figure 2:**
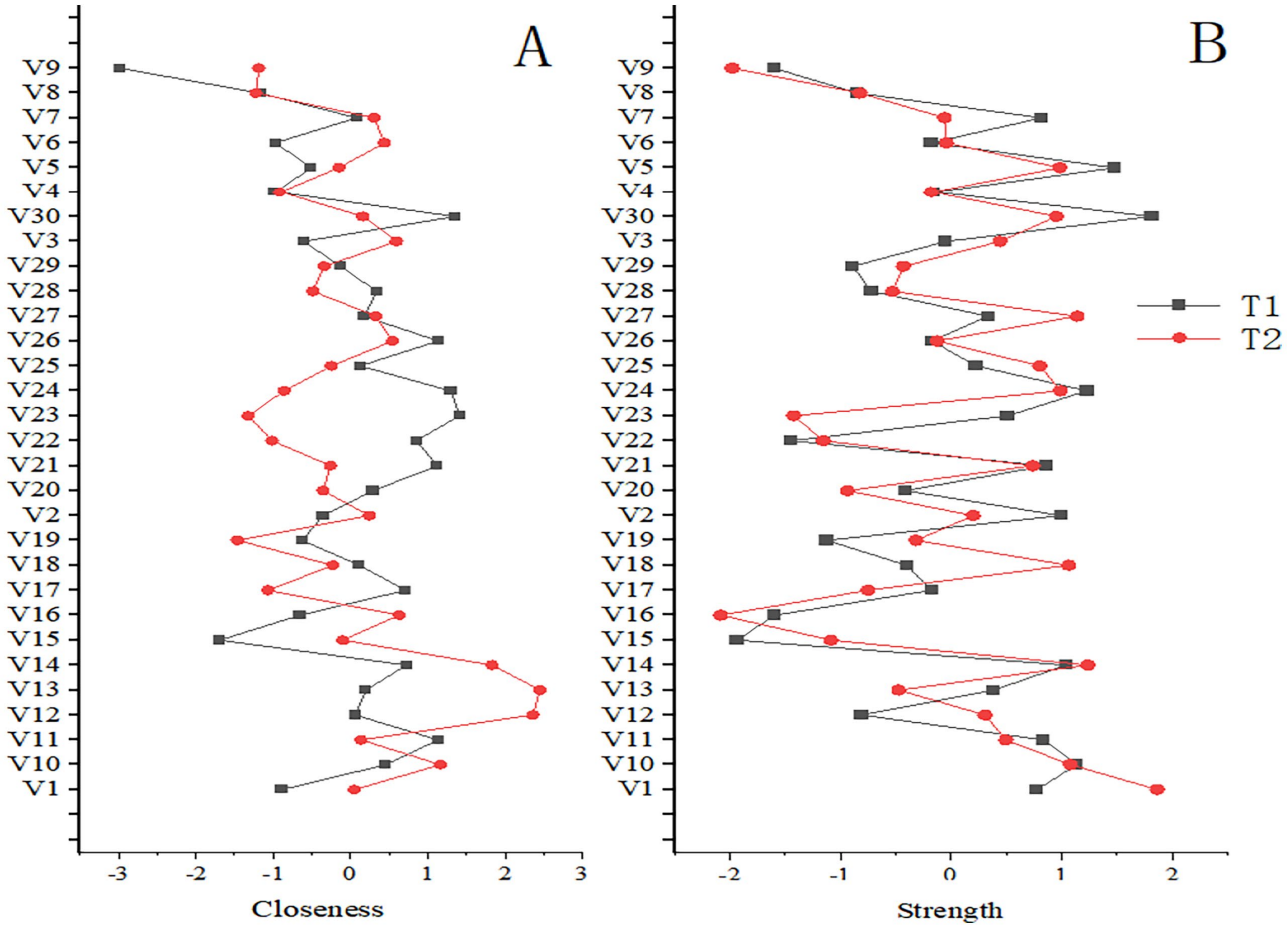
Network centrality metrics for the cross-sectional analysis of T1 and T2. A = closeness; B = strength. Black squares represent T1, and red circles represent T2. V1 = Hopeful, V2 = Enjoyable, V3 = Interesting, V4 = Friendly, V5 = Rewarding, V6 = Worthwhile, V7 = Full, V8 = Brings out the best in me, V9 = Satisfied, V10 = Hard to wind down, V11 = Over-react, V12 = Drain nervous energy, V13 = Agitated, V14 = Difficult to relax, V15 = Intolerant, V16 = Touchy, V17 = Dryness of my mouth, V18 = Breathing difficulty, V19 = Trembling, V20 = Worried fool, V21 = Panic, V22 = Heart rate increase or missing a beat, V23 = Scared, V24 = Lack of positive felling, V25 = Absence of initiative, V26 = Nothing to look forward, V27 = Down hearted and blue, V28 = Unable to be enthusiastic, V29 = Useless, V30 = Life was meaningless.

Together, the descriptive density values and the NCT results suggested that the T1 and T2 networks exhibited broadly similar structures. Within-community connections were characterized by strong positive partial correlations (represented by blue edges) among the SWB nodes and among the ED nodes, respectively. In contrast, the between-community connections linking the SWB and ED item clusters were predominantly negative (represented by red edges), reflecting the expected inverse relationships between specific ED symptoms and facets of SWB. Additionally, the thickness of the edges reflects the magnitude of the association. Thicker lines indicate stronger statistical relationships between the nodes. However, because the comparison was based on two cross-sectional networks, the results should be interpreted as evidence of structural similarity rather than evidence of stable causal dynamics (Figure 2).

Centrality indices, including strength and closeness, were estimated to identify the most central nodes in the T1 and T2 cross-sectional networks. Strength reflects the sum of the absolute weights of all edges directly connected to a node and indicates how strongly that node is directly linked to the rest of the network. Closeness is the inverse of the total shortest-path distance from a node to all other nodes in the network, indicating how close a node is to them.

For strength, Life was meaningless (V30) showed the highest value at T1 (1.820). At T2, Hopeful (V1) showed the highest strength value (1.868). These results indicate that the nodes with the strongest direct connections changed across the two waves.

For descriptive purposes, Scared (V23) showed the highest closeness value at T1 (1.410), whereas Agitated (V13) showed the highest value at T2 (2.450). Given the methodological concerns regarding the stability and interpretability of closeness in psychological networks, these results are reported only as exploratory descriptive information. No substantive conclusions were based on the closeness rankings.

### 3.6 Cross-lagged network analysis

**Figure 3:**
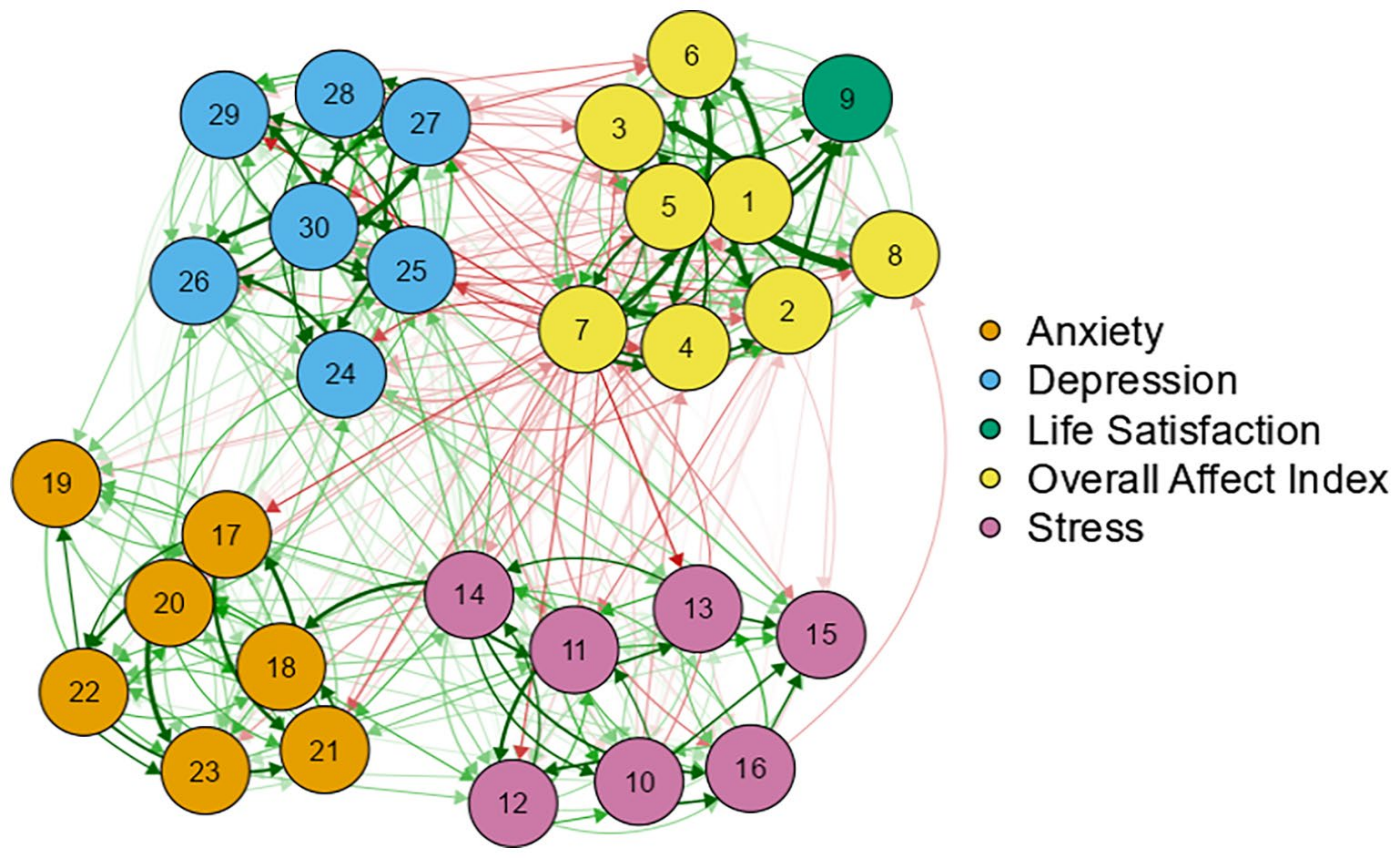
Analysis of the cross-lagged network between T1–T2 emotional distress and subjective wellbeing. 1 = Hopeful, 2 = Enjoyable, 3 = Interesting, 4 = Friendly, 5 = Rewarding, 6 = Worthwhile, 7 = Full, 8 = Brings out the best in me, 9 = Satisfied, 10 = Hard to wind down, 11 = Over-react, 12 = Drain nervous energy, 13 = Agitated, 14 = Difficult to relax, 15 = Intolerant, 16 = Touchy, 17 = Dryness of my mouth, 18 = Breathing difficulty, 19 = Trembling, 20 = Worried fool, 21 = Panic, 22 = Heart rate increase or missing a beat, 23 = Scared, 24 = Lack of positive felling, 25 = Absence of initiative, 26 = Nothing to look forward, 27 = Down hearted and blue, 28 = Unable to be enthusiastic, 29 = Useless, 30 = Life was meaningless. Autoregressive paths were included in the cross-lagged panel network model. To improve readability, the main figure highlights cross-lagged paths, and the full model, including autoregressive paths, is presented in the Supplementary material.

The cross-lagged network between SWB and ED from T1 to T2 showed bidirectional predictive relations between the two domains across the 1-year interval (Figure 3). In the network, green edges represent positive predictive associations, whereas red edges represent negative predictive effects. We further reported the strongest cross-domain predictive edges from T1 to T2. The strongest SWB-to-ED path was T1 Full predicting T2 Agitated, with a coefficient of −0.064. The strongest ED-to-SWB path was T1 Unable to be enthusiastic predicting T2 Friendly, with a coefficient of −0.047. Although these were the strongest cross-domain estimates in the regularized network, both coefficients were small in absolute magnitude. They therefore indicate relatively stronger conditional predictive pathways within the estimated network rather than large longitudinal effects. These paths should be interpreted as regularized longitudinal predictive associations rather than causal effects.

**Figure 4:**
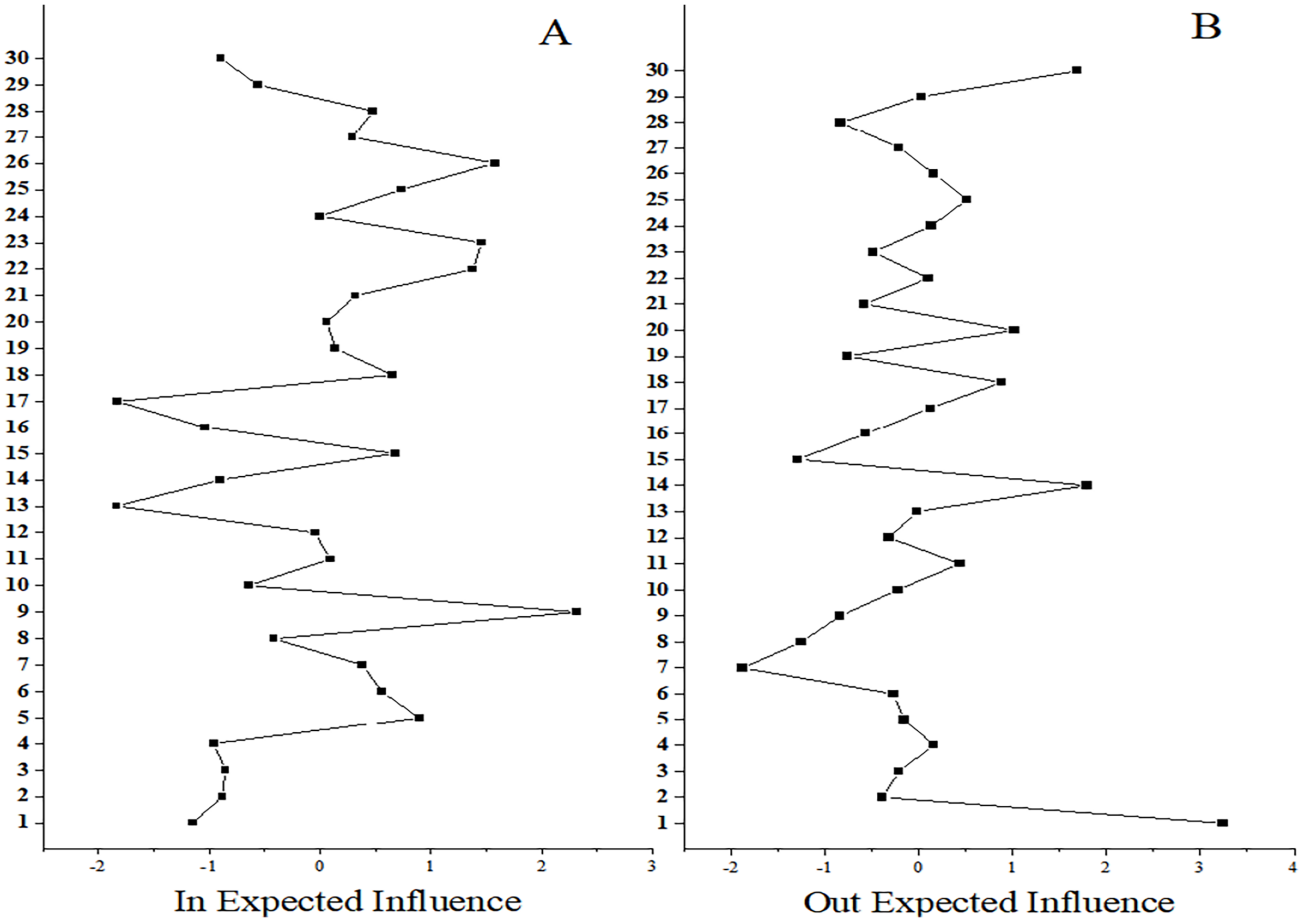
Cross-lagged network centrality metrics. A = in-expected influence; B = out-expected influence. 1 = Hopeful, 2 = Enjoyable, 3 = Interesting, 4 = Friendly, 5 = Rewarding, 6 = Worthwhile, 7 = Full, 8 = Brings out the best in me, 9 = Satisfied, 10 = Hard to wind down, 11 = Over-react, 12 = Drain nervous energy, 13 = Agitated, 14 = Difficult to relax, 15 = Intolerant, 16 = Touchy, 17 = Dryness of my mouth, 18 = Breathing difficulty, 19 = Trembling, 20 = Worried fool, 21 = Panic, 22 = Heart rate increase or missing a beat, 23 = Scared, 24 = Lack of positive felling, 25 = Absence of initiative, 26 = Nothing to look forward, 27 = Down hearted and blue, 28 = Unable to be enthusiastic, 29 = Useless, 30 = Life was meaningless.

To evaluate node importance in the longitudinal network, this study examined in-expected influence and out-expected influence. In-expected influence reflects the extent to which the T1 network predicts a T2 node, whereas out-expected influence reflects the extent to which a T1 node predicts other nodes at T2. The highest in-expected influence value appeared for Satisfied (Martela and Ryan, 2023), with a value of 2.31, which indicates that this node was the most strongly predicted by the overall T1 network (Figure 4A). The highest out-expected influence value appeared for Hopeful (Wang et al., 2020), with a value of 3.24, which suggests that this node had the strongest outgoing predictive connectivity to other nodes at T2 (Figure 4B).

Taken together, the cross-lagged results indicate that Hopeful (V1) showed the highest outgoing predictive connectivity in the longitudinal network, whereas Satisfied (V9) was the node most strongly predicted by the prior network structure.

### 3.7 Network stability

Network stability was evaluated with a case-drop bootstrap procedure. The correlation stability coefficient (CS-coefficient) serves as the main index of stability. In general, a CS-coefficient above 0.25 indicates acceptable stability, whereas a value above 0.50 indicates good stability (Zhang et al., 2025). In this study, the CS (cor = 0.7) was 0.52.

**Figure 5:**
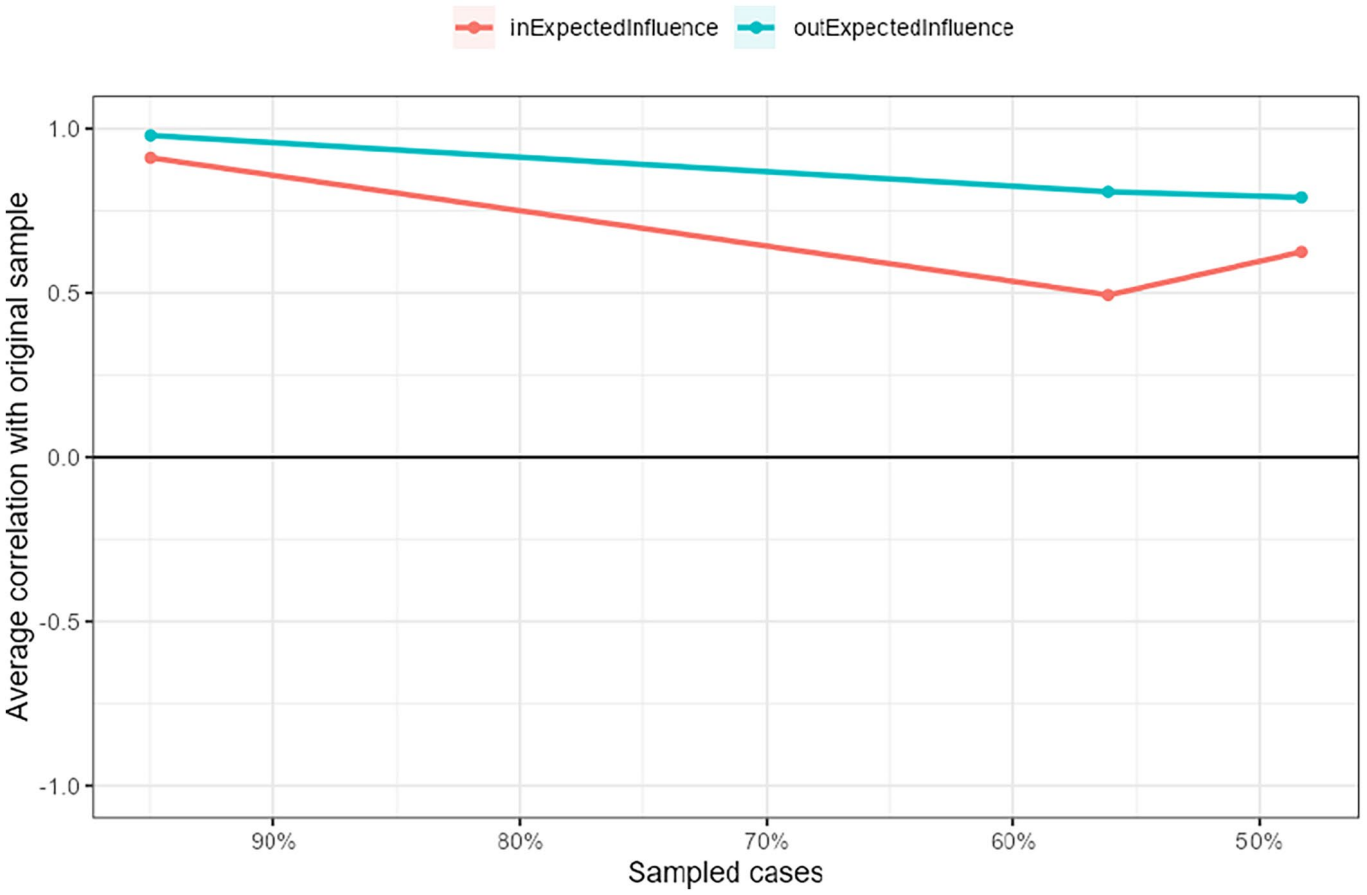
Stability of centrality measures in cross-lagged network analysis.

As shown in Figure 5, both in-expected influence and out-expected influence maintained relatively high correlations with the original sample across different sampling proportions. The out-expected influence curve remained more stable overall, whereas the in-expected influence curve showed a greater decline as the sampled cases decreased. Overall, the results suggest that the centrality estimates of the cross-lagged network were sufficiently stable for interpretation.

**Figure 6:**
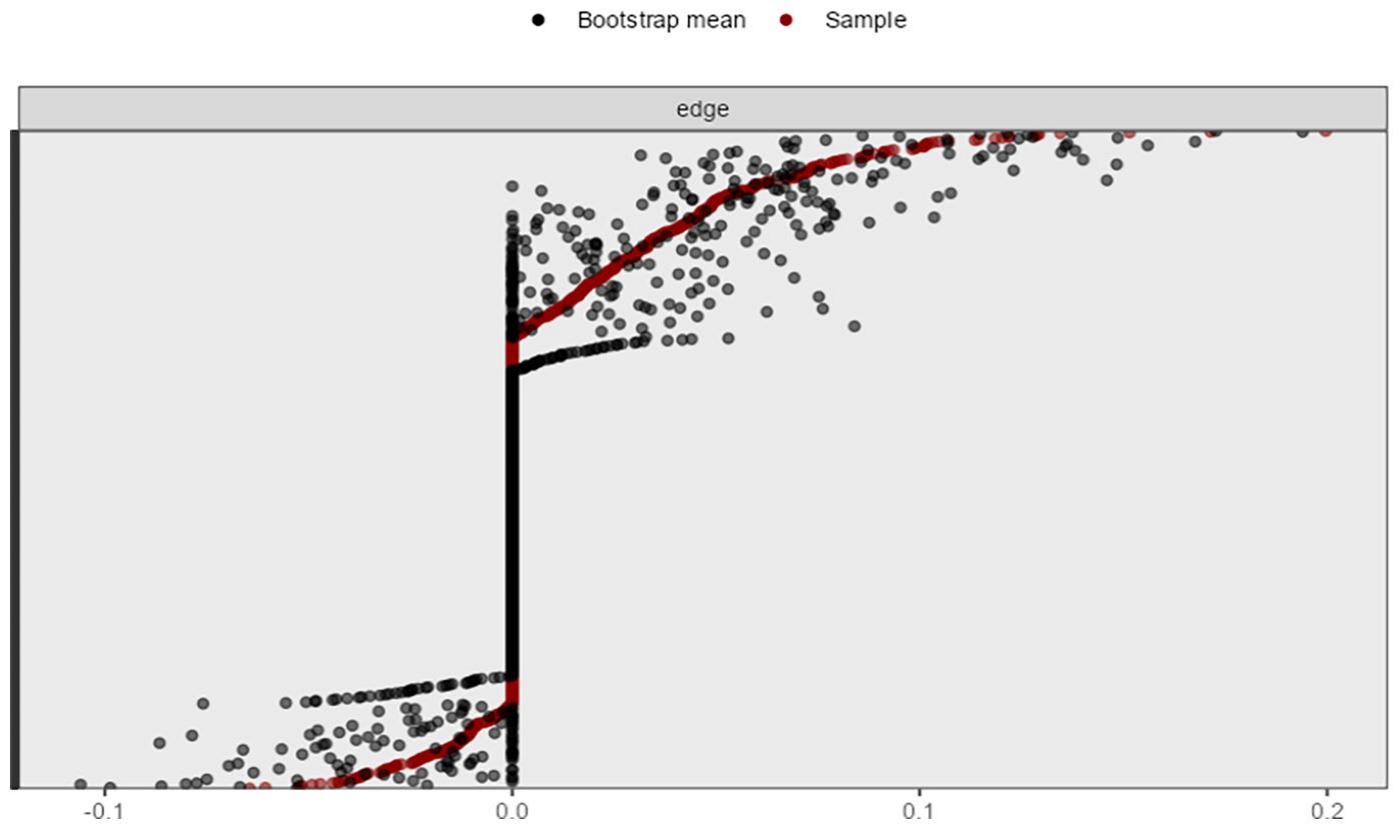
Accuracy test for edge weights in cross-lagged network analysis.

**Figure 7:**
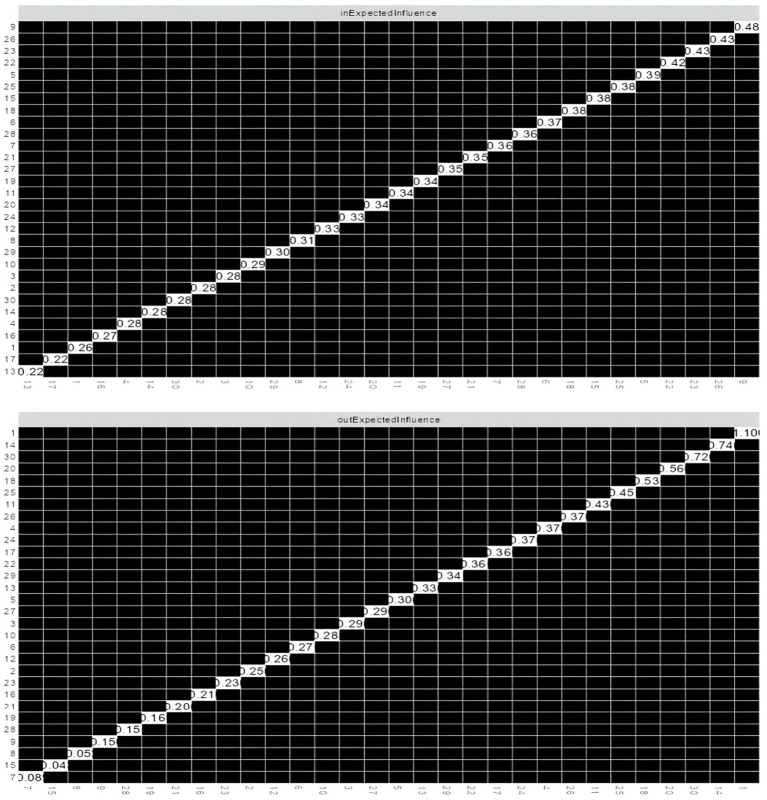
Centrality-difference tests in cross-lagged network analysis.

A non-parametric bootstrap procedure with 1,000 iterations was used to assess the accuracy of the edge-weight estimates. Figure 6 showed that most estimated edge weights were centered around the sample estimates, suggesting acceptable precision for the main edges. Some weak edges had wider intervals or were close to zero, so they were interpreted with caution. Bootstrap-based centrality-difference tests further indicated that Hopeful, V1, and Satisfied, V9, ranked relatively high in out-expected influence and in-expected influence, respectively, compared with many lower-ranked nodes (see Figure 7). These findings support the robustness of the main centrality pattern, but they do not imply causal priority.

## 4 Discussion

The present findings should be interpreted within a dual-factor framework of mental health. From this perspective, SWB and ED are not simply opposite ends of a single continuum; rather, they are related yet partly separable systems that can influence each other at the component level. The cross-sectional networks support this view, as SWB items and ED items were strongly connected within their respective domains. At the same time, several negative between-domain edges showed that positive functioning and distress symptoms were also linked across domains. Thus, the key implication is not only that SWB and ED are associated, but that their association may operate through specific cognitive, affective, and arousal-related components. The cross-lagged network showed bidirectional predictive relations from T1 to T2, suggesting that SWB and ED did not function as two isolated systems. This overall pattern is consistent with earlier longitudinal research showing predictive associations between wellbeing and emotional distress (Conley et al., 2023; Pellerin et al., 2024; Groarke et al., 2020; Shanahan et al., 2020). However, the strongest cross-domain coefficients, −0.064 and −0.047, were small in absolute magnitude. These estimates identify the strongest cross-domain pathways in the current regularized network but should not be interpreted as evidence of large longitudinal effects. Their practical significance remains uncertain and should be evaluated through replication in independent longitudinal samples.

Within this structure, the prominent cross-sectional nodes may reflect how college students’ wellbeing and distress are organized around meaning-related cognition and arousal-related symptoms. At T1, Life was meaningless showed the highest strength. This may be because perceived meaning is closely tied to students’ evaluation of life value, future direction, and self-worth (Cockle and Schnitker, 2024). When students feel that life lacks meaning, this belief may co-occur with depressive cognitions, such as uselessness, lack of enthusiasm, and lack of positive feeling, while also weakening positive evaluations such as hopefulness, satisfaction, and worthwhileness (Hou et al., 2025). This explanation is consistent with Beck’s cognitive theory, which emphasizes the role of negative beliefs in emotional symptoms (Giles and Shaw, 1987), and with previous studies showing that meaning in life is negatively associated with depression, anxiety, and psychological distress (Cai et al., 2024; Dong et al., 2025; Wang et al., 2020; Steger et al., 2009; Yuan et al., 2025). Compared with these variable-level studies, the present network result provides more specific evidence by showing that meaning-related cognition occupied a highly connected position at the item level.

The longitudinal network extends this interpretation by suggesting different predictive roles for Hopeful and Satisfied. Hopeful showed the highest outgoing predictive connectivity, which may reflect the role of future-oriented expectations in agency and pathway thinking. Students who feel hopeful may be more likely to view academic and interpersonal difficulties as manageable, a tendency that could be associated with later positive affect and lower distress (Rodriguez et al., 2020; Wong and Cheung, 2024; Rand et al., 2020); however, this remains a plausible mechanism rather than a causal conclusion because the study did not manipulate hope or directly measure coping behavior. Satisfied showed the highest incoming predictive connectivity, which is consistent with life satisfaction as a broad evaluative judgment. Rather than being treated as a final causal outcome, Satisfied may be understood as an integrative indicator that summarizes prior emotional symptoms, positive affective experiences and broader evaluations of life circumstances (Tian et al., 2021; Lee, 2024; Feraco et al., 2022; Chang et al., 2019; Umucu et al., 2022).

## 5 Implications

This study extends the growing network-based literature on wellbeing and emotional distress by providing additional item-level evidence from a two-wave sample of college students. Consistent with previous symptom-level and network-based research, the findings suggest that SWB and ED are connected through specific cognitive, affective, and physiological components rather than only through global construct scores. The present results add a longitudinal perspective by identifying item-level predictive associations across a 1-year interval.

A second implication concerns the role of hope. The item “Hopeful” showed the strongest out-expected influence in the cross-lagged network, indicating that it had relatively high outgoing predictive connectivity with later network states. This finding does not show that changing hope would produce the largest improvement in student mental health. It does, however, suggest that future studies should examine hope-related cognition more closely, particularly because hope reflects future-oriented expectations, perceived possibilities, and goal-related thinking. Intervention research in higher education may therefore test whether structured approaches that strengthen hopeful thinking, such as goal clarification, pathway planning, and agency-based exercises, are associated with later improvements in wellbeing or reductions in distress.

Finally, the finding that “Satisfied” showed the strongest in-expected influence suggests that life satisfaction may serve as a broad summary indicator of prior psychological states. Because life satisfaction is an evaluative judgment, it may reflect the combined influence of earlier wellbeing and distress components. This does not mean that life satisfaction should be treated as a stand-alone outcome or diagnostic marker. Instead, it may be useful as part of a broader monitoring framework that also includes distress symptoms, meaning-related cognition, and future-oriented hope. For universities, this finding supports a more fine-grained approach to mental health assessment, in which general wellbeing indicators are interpreted together with specific symptom-level and resource-level information.

## 6 Limitations

Several limitations should be noted. First, the study used a convenience sample from six universities in three provinces of China. Although the sample size was adequate, it was limited to six universities across three provinces of China. Students from other regions, types of universities, cultural contexts, or non-college populations may exhibit different patterns. Therefore, the findings should be generalized with caution. Future studies should include more diverse samples to test the robustness of the present findings.

Second, all data were collected via self-report questionnaires using similar response procedures, which may have introduced response bias and shared method variance. Although the first factor in Harman’s single-factor test accounted for less than 40% of the variance at both waves, this procedure provides only a limited diagnostic assessment and cannot exclude common method variance. In addition, because the survey link was distributed via class-based online channels, the study was unable to determine the exact number of students who received or opened it at each university. Consequently, precise university-level response rates and comparisons between participants and non-participants could not be calculated. Additionally, participation was voluntary, which may have introduced self-selection bias. Students who were more interested in mental health topics or more willing to complete online surveys may have been more likely to participate. Future research may combine self-report data with behavioral, clinical, or interview-based measures to provide a more comprehensive assessment of SWB and ED.

Third, the study included only two waves of data, with a 1-year interval between them. The interval was chosen to capture changes across an academic year, but this timescale may not be optimal for all components of wellbeing and distress. Students may experience many unmeasured contextual changes over the course of a year, including academic progression, internships, examination preparation, employment-related stressors, and changes in social relationships. Although the cross-lagged network analysis identified bidirectional predictive relations across time, a two-wave design cannot capture feedback processes, nonlinear developmental trajectories, or short-term fluctuations. Future studies should include three or more waves, shorter assessment intervals, and contextual measures to examine temporal dynamics in greater detail.

Fourth, attrition should also be considered. Of the 1,597 valid T1 participants, 1,354 were included in the final matched sample, indicating an attrition rate of approximately 15.2%. We were unable to compare retained participants with those lost to follow-up because the unmatched baseline records were discarded after wave matching. This decision followed a data-minimization approach to protect participant privacy. After T1-T2 matching was completed, the linkage information and records that could not be incorporated into the de-identified longitudinal analytic dataset were removed from the working dataset. Consequently, the direction and magnitude of potential attrition bias remain unknown. It cannot be determined whether the loss of participants resulted in an overestimation, underestimation, or other systematic alteration of the reported associations. No imputation or weighting adjustment was applied, and the CLPN analyses were conducted only among participants with valid matched data at both waves. Future studies should retain de-identified baseline variables for both retained and lost participants, with appropriate consent and data-governance procedures, so that the direction and magnitude of attrition bias can be formally evaluated.

Fifth, the present study focused only on SWB and ED. Other factors, such as social support, academic stress, coping style, and personality traits, may also influence the network structure and the longitudinal relations between nodes. Future research may incorporate these variables to clarify the broader context in which SWB and ED develop.

## 7 Conclusion

The present study examined the relationship between SWB and ED among college students from a network perspective. The cross-sectional networks showed that the two domains were closely connected at both time points, with predominantly positive associations within each domain and predominantly negative associations between them. In the longitudinal network, SWB and ED showed bidirectional predictive relations across the 1-year interval. Among all nodes, Hopeful showed the strongest out-expected influence, whereas Satisfied showed the strongest in-expected influence. These findings suggest that SWB and ED are interconnected at the symptom level among college students. Overall, the study provides a cautious symptom-level account of how positive and negative aspects of mental health may be related over time.

## Data Availability

The raw data supporting the conclusions of this article will be made available by the authors, without undue reservation.
