# Supplementary figures and images for: The bidirectional relationship between emotional distress and subjective wellbeing among college students: a cross-lagged network analysis
Source: Front Psychol. 2026 Jul 6;17:1863736. doi: 10.3389/fpsyg.2026.1863736 (PMC13383040; doi:10.3389/fpsyg.2026.1863736)

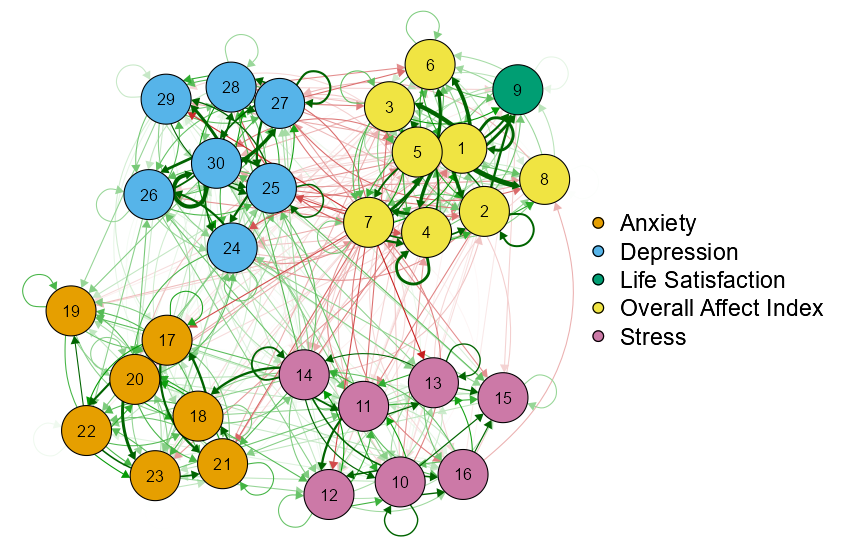

Supplement: Supplementary file 1 [file Image_1.png]
